# Development of a questionnaire to assess sedentary time in older persons – a comparative study using accelerometry

**DOI:** 10.1186/1471-2318-13-80

**Published:** 2013-07-30

**Authors:** Marjolein Visser, Annemarie Koster

**Affiliations:** 1 Department of Health Sciences, Faculty of Earth and Life Sciences, VU University Amsterdam, De Boelelaan 1085, 1081HV, Amsterdam, the Netherlands; 2 Department of Epidemiology and Biostatistics, EMGO Institute for Health and Care Research, VU University Medical Center, Amsterdam, the Netherlands; 3 Department of Social Medicine, CAPHRI School for Public Health and Primary Care, Maastricht University, PO Box 616, 6200MD, Maastricht, the Netherlands

**Keywords:** Sedentary behavior, Accelerometry, Physical activity, Aging

## Abstract

**Background:**

There is currently no validated questionnaire available to assess total sedentary time in older adults. Most studies only used TV viewing time as an indicator of sedentary time. The first aim of our study was to investigate the self-reported time spent by older persons on a set of sedentary activities, and to compare this with objective sedentary time measured by accelerometry. The second aim was to determine what set of self-reported sedentary activities should be used to validly rank people’s total sedentary time. Finally we tested the reliability of our newly developed questionnaire using the best performing set of sedentary activities.

**Methods:**

The study sample included 83 men and women aged 65–92 y, a random sample of Longitudinal Aging Study Amsterdam participants, who completed a questionnaire including ten sedentary activities and wore an Actigraph GT3X accelerometer for 8 days. Spearman correlation coefficients were calculated to examine the association between self-reported time and objective sedentary time. The test-retest reliability was calculated using the intraclass correlation coefficient (ICC).

**Results:**

Mean total self-reported sedentary time was 10.4 (SD 3.5) h/d and was not significantly different from mean total objective sedentary time (10.2 (1.2) h/d, p = 0.63). Total self-reported sedentary time on an average day (sum of ten activities) correlated moderately (Spearman’s r = 0.35, p < 0.01) with total objective sedentary time. The correlation improved when using the sum of six activities (r = 0.46, p < 0.01), and was much higher than when using TV watching only (r = 0.22, p = 0.05). The test-retest reliability of the sum of six sedentary activities was 0.71 (95% CI 0.57-0.81).

**Conclusions:**

A questionnaire including six sedentary activities was moderately associated with accelerometry-derived sedentary time and can be used to reliably rank sedentary time in older persons.

## Background

In the past few years there has been a growing interest in the health effects of sedentary behavior. Estimates of self-reported sedentary behavior, such as television (TV) viewing time, were found to be positively associated with cardio-metabolic risk factors and disease and mortality [1-3].

Sedentary behavior is most common in older persons, compared to any other age group [4-6]. Based on objective measurements of physical activity using accelerometry, total sedentary time in adults aged 60–75 years living in Sweden and the United States was 8.4 and 9.0 hours a day, or about 59 to 65% of wear time, respectively [5]. Total sedentary time for older men and women (age 73–98 years) living in Iceland was about 75% of their wear time [7].

Accurate assessment of sedentary time in addition to physical activity time is important and may contribute to our understanding of how sedentary behavior may influence healthy aging. Most studies investigating the association between self-reported sedentary behavior and health outcomes have used TV viewing time as an indicator of sedentary behavior. Although studies have shown that a substantial part of sedentary time is indeed spent watching TV [8-10], two recent reviews concluded that sedentary behavior should not be limited to TV viewing but should include a broad range of sedentary activities [11,12]. The authors also referred to the need to develop reliable and valid questionnaires to determine sedentary behavior. Furthermore, most of the research on sedentary activity has been performed in children and young adults, while less research has been done in older adults.

The first aim of this study was to investigate the self-reported time spent by older persons on multiple sedentary activities, and how this relates to objective sedentary time as measured by tri-axial accelerometry. The second aim was to determine what set of sedentary activities should be used to validly rank older people’s sedentary time, using accelerometry as the reference method. Finally, we tested the reliability of our newly developed questionnaire containing the best performing set of sedentary activities.

## Methods

### Study participants

Data for this study were collected in the context of the Longitudinal Aging Study Amsterdam (LASA). The sampling and data collection procedures and the response rates have been described in detail elsewhere [13]. Briefly, a random sample stratified by age, sex, and expected 5-year mortality was drawn from the population registers of 11 municipalities in three geographical areas in the west, north-east, and south of the Netherlands. A total of 3107 subjects were enrolled in the baseline examination (1992–1993) and were representative of the Dutch older population. In 2002–2003, a new cohort of 1002 men and women aged 55–65 years was added to the study using the same sampling procedures. Informed consent was obtained from all participants. The study was approved by the Medical Ethics Committee of the University Medical Center, VU University Amsterdam.

In April 2010, a random sample of 130 persons were sent an information letter and were contacted by phone to ask them to participate in a sub-study on sedentary behavior. Ninety-three of these men and women agreed to participate in the study. Reasons for non-response were death (n = 2), refused (n = 17), no contact (n = 8), too ill or frail (n = 5), holiday (n = 4), and unknown (n = 1). Questionnaire and accelerometry data were obtained from 88 participants. We then selected participants with at least 1 valid day of accelerometry data and a valid weekday or weekend day score on the questionnaire. This left 83 participants aged 65 to 92 years for the current analysis.

### Self-reported sedentary time

Together with the accelerometer, a sealed envelope was mailed to the participants, with the instruction to open it after the final day of wearing the device. The envelope contained a self-administered questionnaire regarding the time spent on ten different sedentary activities (Additional file 1). The questionnaire consisted of sedentary items that were adapted from previous questionnaires developed for younger adults and children [14-17]. Participants were instructed to report the time they generally spent on each sedentary activity per day. The ten items had to be completed for a regular weekday as well as for a regular weekend day. The activities included ‘taking a nap during the day’ (napping), ‘reading’ (reading), ‘listening to music while sitting or lying down’ (listening to music), ‘watching TV, video or DVD’ (watching TV), ‘sitting at the computer for work or leisure’ (computer), ‘performing seated activities such as administrative work, writing a letter or having a meeting’ (working), ‘performing hobbies while being seated, such as playing a musical instrument, doing jigsaw puzzles or knitting’ (hobby), ‘talking (on the phone) to family, friends or acquaintances’ (talking), ‘sitting in a car, bus or train’ (transportation), ‘visiting church or theater (including cinema)’ (church/theater). Participants were instructed to only report one activity when they had performed two or more activities at the same time: ‘if you performed two activities at the same time, for example listening to music while knitting, please report only one of the two activities. It’s up to you to decide for which activity you want to report this time.’ The completed questionnaire was mailed back to the researchers together with the accelerometer. After 23 (SD 8) days, the questionnaire was mailed to the participants again, to assess its reliability. Sixty-three participants returned both completed questionnaires. Total self-reported sedentary time on weekdays and weekend days were calculated by adding up the time spent on all six sedentary activities. The total score was considered missing (invalid) when more than four items were missing.

### Objective measurement of sedentary time

The Actigraph triaxial accelerometer (Model GT3X; Actigraph Inc., Pensacola, FL) was used to objectively measure sedentary time. The accelerometer, together with an instruction brochure which included photographs of how to properly wear the accelerometer, was sent to the participants by regular mail. After two days, they were phoned to ensure that the package had been received and the accelerometer was being properly worn. The accelerometer was attached to a 3 cm wide, tight elastic belt and was worn around the waist above the left iliac crest. Participants were briefed to wear the accelerometer for an 8-day period during waking hours and to return the accelerometer in the envelope provided. Activity was recorded using 1-second epochs, which were added up to minute-to-minute epochs. Accelerometry data were obtained from 91 participants.

The data were processed using customized software written in MATLAB R2006a (The MathWorks, Inc., Natick, MA). Nonwear time was defined as a 60-minute window of zero counts in all three axes, allowing for up to two minutes of nonzero counts <100 in the vertical axis. Data files with fewer than 10 hours per day of wear time were excluded [18]. The majority of the study sample (78%) had eight valid accelerometry days. The total objective sedentary time per day was assessed using the <100 counts per minute cut-off point based on the vertical axis [6,19]. Total objective sedentary time during weekdays and weekend days was calculated using all valid days of monitoring during those days.

### Other variables

The following variables were obtained from the regular LASA measurement cycle conducted in 2008–2009: sex, date of birth, education level, body weight and body height. The participant’s age on the first day of wearing the accelerometer was calculated. Level of education was categorized as low (primary school or less), medium, and high (higher vocational, college or university education). Body weight was measured without clothes or shoes, using a calibrated scale. Body height was measured using a stadiometer. The body mass index (BMI) was calculated as body weight in kilograms divided by height in meters squared.

### Statistical analysis

Potential differences between the study sample and the overall LASA sample were tested using Student’s t-test for continuous variables and Chi-square test for categorical variables. Total self-reported sedentary time and total objective sedentary time for an average day were calculated as ((total sedentary time on weekdays * 5) + (total sedentary time on weekend days * 2))/7. Four participants had data for weekdays only or weekend days only, and their sedentary time on an average day was based on those days only. Differences between weekdays and weekend days in self-reported time spent on each individual sedentary activity and total self-reported sedentary time were tested using paired Student’s t-tests. Potential differences between men and women were tested with Student’s t-test. The difference between total self-reported sedentary time and total objective sedentary time was tested using a paired t-test. As the distribution of self-reported time spent on individual sedentary activities was skewed for most activities, Spearman correlation coefficients were calculated to examine the association between self-reported time and total objective sedentary time. To determine which set of sedentary activities allowed an optimal ranking of older men and women with regard to sedentary time, Spearman correlation coefficients were calculated for the summed self-reported times of all possible combinations of sedentary activities and total objective sedentary time from accelerometry. Spearman correlation coefficients were also calculated separately for weekdays and weekend days in sensitivity analyses.

The Bland–Altman method was used to determine the level of agreement between the best performing set of self-reported sedentary activities and objective sedentary time and to determine potential variations in this agreement over the range of measurement [20]. The test-retest reliability was investigated by calculating the intraclass correlation coefficient (ICC) using a two-way random effects model. An ICC of at least 0.70 was considered sufficient reliability. All analyses were conducted using SPSS version 17.0 (SPSS Inc., Chicago, IL). P-values were based on two-sided tests and considered statistically significant if less than 0.05.

## Results

General characteristics of the study sample are shown in Table 1. Compared to the overall LASA sample, the study sample was similar with regard to sex, age and body mass index, but had a higher education level.

**Table 1:** Characteristics of the participants with complete questionnaire and accelerometry data on sedentary time

	**Sedentary behavior sample**	**Overall LASA sample**	**p-value**
N	83	1735	
Female (%)	49.4	57.0	0.17
Age (years), mean (SD)	74.3 (6.9)	73.9 (9.0)	0.97
Education level (%)			
Low	13.3	28.2	0.012
Medium	65.1	54.1	
High	21.7	17.7	
BMI (kg/m^2^), mean (SD)	27.0 (3.7)	27.5 (4.4)	0.23

### Self-reported sedentary time

**Figure 1:**
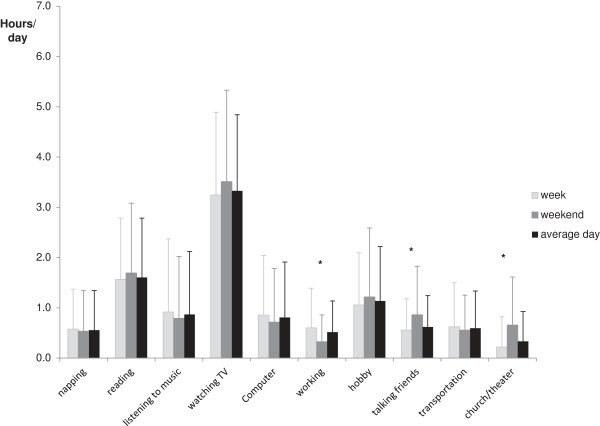
**The mean self-reported time (h/d, with standard deviation) spent on 10 sedentary activities for weekdays, weekend days and an average day.** *P < 0.05 week versus weekend day.

Figure 1 shows the mean self-reported time spent on each sedentary activity addressed in the questionnaire for weekdays and weekend days. On weekend days, participants spent relatively more time talking (p < 0.01) and visiting a church/theater (<0.01) and less time on work (p < 0.01) and computer activities (p = 0.08). The mean time spent on each of the activities on an average day of the week is also shown in Figure 1. Most sedentary time was reported while watching TV (3.3 h/d), followed by reading (1.6 h/d) and hobby (1.1 h/d).

Total self-reported sedentary time tended to be lower on weekdays (10.2 h (SD 3.8)) than on weekend days (10.8 h (SD 3.5), p = 0.06) (Table 2). No differences were observed between men and women (p = 0.87), but some gender differences in individual sedentary activities were observed. On weekdays and average days, women spent more time on hobbies (p < 0.05), talking (p = 0.02), and church/theater visits (p = 0.04) than men. On weekend days, men spent more time watching TV (p = 0.02).

**Table 2:** Mean subjective sedentary time and objective sedentary time by accelerometry and other accelerometry variables

	**Weekday Mean (SD)**	**Weekend day Mean (SD)**	**p**	**Average day **^**a **^**Mean (SD)**
**Questionnaire**				
Sedentary time (hours)	10.2 (3.8)	10.8 (3.5)	0.06	10.4 (3.5)
**Accelerometry**				
Valid days, n	5.4 (1.2)	2.1 (0.6)	-	7.5 (1.5)
Mean wear time (hours)	15.3 (1.1)	14.9 (1.4)	<0.01	15.1 (1.1)
Mean counts per minute	227.6 (145.4)	198.2 (111.8)	<0.01	219.5 (130.7)
Sedentary time (hours)	10.1 (1.2)	10.3 (1.4)	0.08	10.2 (1.2)
Relative sedentary time (%)	66.4 (8.7)	69.6 (8.1)	<0.01	67.3 (8.2)

^a^ Average day = (weekday*5 + weekend day*2)/7.

### Objectively measured sedentary time

Table 2 also shows the results of the objective accelerometer data. The mean total objective sedentary time was 10.1 (SD 1.2) hours on a weekday, 10.3 (SD 1.4) hours on a weekend day, and 10.2 (SD 1.2) hours on an average day of the week. Total objective sedentary time tended to be lower on weekdays than on weekend days (p = 0.08). Relative sedentary time (total sedentary time divided by total wear time) was significantly higher on weekend days compared to weekdays (p < 0.01). No differences between men and women were observed in any of the accelerometry parameters (p > 0.65).

### Comparing self-reported and objective sedentary time

Total self-reported sedentary time was not significantly different from total objective sedentary time (p = 0.63). The Spearmen correlation coefficients of the relationship between mean self-reported time spent on ten sedentary activities and total objective sedentary time are shown in Table 3. The strongest, but still non-significant, positive associations with total objective sedentary time (r > 0.20) were observed for reading, watching TV and hobby. Weaker positive associations (r = 0.10-0.19) were found for napping and listening to music. The time spent sitting in a car, bus or train and the time spent sitting in church or theater were inversely correlated with the total objective sedentary time. The correlations between the individual sedentary activities are also shown in Table 3.

**Table 3:** Spearman correlation coefficients of self-reported time spend on sedentary activities and total objective sedentary time

	**Napping**	**Reading**	**Listening to music**	**Watching TV**	**Computer**	**Working**	**Hobby**	**Talking friends**	**Transportation**	**Church/theater**	**Objective sedentary time**
Napping	1.00										
Reading	0.04	1.00									
Listening to music	−0.004	0.31*	1.00								
Watching TV	−0.08	0.05	−0.02	1.00							
Computer	−0.23*	0.07	−0.08	0.14	1.00						
Working	−0.02	0.27*	0.29*	−0.01	0.24*	1.00					
Hobby	−0.06	−0.04	0.03	0.02	0.09	0.05	1.00				
Talking friends	−0.11	0.11	0.03	0.06	0.13	0.30*	0.08	1.00			
Transportation	0.01	−0.10	−0.08	0.07	−0.02	0.17	−0.09	0.12	1.00		
Church/theater	0.05	−0.09	0.11	−0.13	−0.04	0.19	−0.07	−0.01	0.19	1.00	
Objective sedentary time	0.11	0.21	0.14	0.22	0.04	0.002	0.20	0.05	−0.06	−0.19	1.00

* Statistically significant p < 0.05.

**Figure 2:**
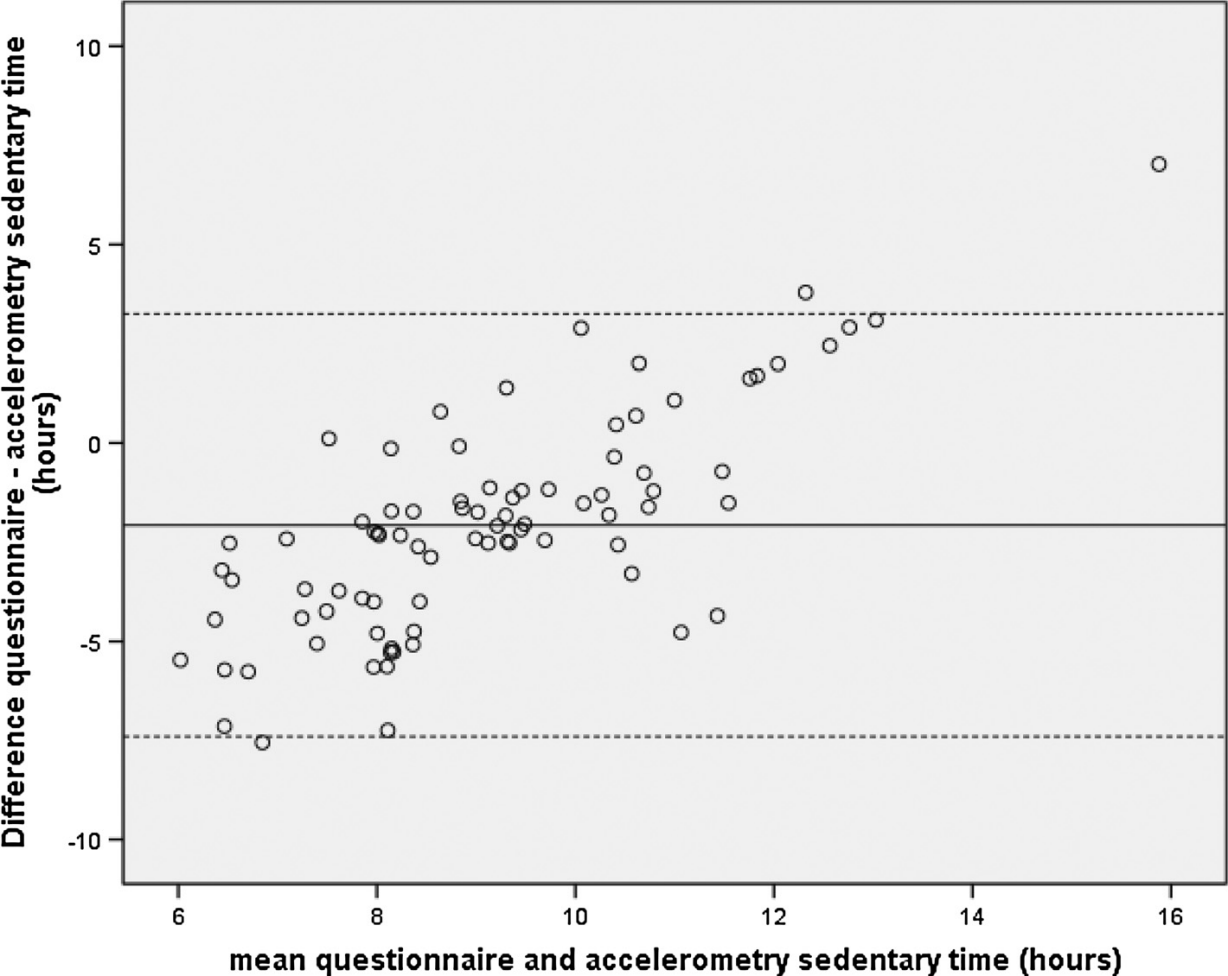
**Bland-Altman plot of the difference between and the mean of self-reported sedentary time based on six activities**^**a **^**and total objective sedentary time. **^a^Included activities were napping, reading, listening to music, watching TV, hobby, and talking with friends.

The Spearman correlation coefficient of the association between total self-reported sedentary time based on all ten sedentary activities and total objective sedentary time was 0.35 (p < 0.05, Table 4). The set of sedentary activities that correlated best (r = 0.46, p < 0.05) with objective sedentary time and could therefore be used to optimally rank people’s total sedentary time consisted of six activities: napping, reading, listening to music, watching TV, hobby, and talking to friends. The Bland–Altman plot (Figure 2) shows, as expected, that total sedentary time was underestimated when using only the set of six activities. The mean difference was 2.1 hours, with limits of agreement of −7.40 to 3.25 hours. The plot also shows that persons with less sedentary time were more likely to underestimate their sedentary time and persons with more sedentary time were more likely to overestimate their sedentary time.

**Table 4:** Total self-reported sedentary time based on different sets of sedentary activities and spearman correlation coefficients of their relationships with total objective sedentary time

	**Total score all 10 items**	**Best score with 6 items **^**a**^	**Watching TV**
Time (hours), mean (SD)	10.4 (3.5)	8.1 (3.0)	3.3 (1.5)
Correlation coefficient	0.35*	0.46*	0.22

^a^ Included activities were napping, reading, listening to music, watching TV, hobby, and talking with friends.

* Statistically significant p < 0.05.

In a sensitivity analysis we investigated the association between the total time spent on the best performing set of sedentary activities and the total objective sedentary time, using the results of a weekday or those of a weekend day instead of an average day. The spearman correlation coefficients were 0.42 and 0.36, respectively.

The test-retest reliability was examined in 63 persons, who did not differ from the total sample with regard to sex, education level, age or body mass index (p > 0.27). The reliability of the best performing set consisting of six sedentary activities was 0.71 (95% CI 0.57-0.81). The total sedentary time was 8.2 (3.1) h/d for the first and 8.2 (3.1) h/d for the second questionnaire (p = 0.999). The reliability for the six individual sedentary activities ranged from 0.31 (talking) to 0.85 (napping) and was 0.84 for TV watching.

## Discussion

This is one of the first studies to relate self-reported time spent by older persons on a set of multiple sedentary activities to objectively assessed sedentary time using accelerometry. Total objective sedentary time was 10.2 hours per day, which was 67.3% of the total accelerometer wear time. The percentage of time spent sedentary was significantly higher during the weekend than on weekdays. A set of six sedentary activities from our newly developed questionnaire showed the highest correlation with total objective sedentary time (r = 0.46) and performed best in terms of ranking people’s total sedentary time. The six sedentary activities included time spent napping, reading, listening to music, watching TV, hobby, and talking to friends.

Our objective estimate of total sedentary time was slightly higher than those reported in some recent studies among persons aged 60–75 y (9.0 h/d and 8.4 h/d (3)) and aged 60–85 y (8.6 h/d (2)), but was similar to the 9.8 h/d observed in a sample of older adults aged 66+ years [21] and similar to those reported for men (10.6 h/d) and women (10.1 h/d) aged 73–98 years living in Iceland [7]. All studies used the same cut-off point of <100 counts per minute to determine sedentary time. These studies showed that older persons spent the majority of wear time on sedentary activities.

Most previous studies investigating sedentary time have used the TV watching time or screen time as an estimate of sedentary time. Although studies have shown that most sedentary time is indeed spent watching TV [8-10], the association between TV watching time and total objective sedentary time seems rather weak. Clark et al. reported a Spearman’s rho of 0.22 between TV viewing time and accelerometer-derived sedentary time among 5738 adults aged 20 years and older [22]. This correlation is identical to that observed in the present study. A French study among 160 persons aged 19–63 y reported an even lower Spearman correlation coefficient of 0.14 [23]. These results clearly suggest that TV viewing is not an optimal indicator of total sedentary time and that additional sedentary activities should be addressed. Even though TV viewing has been strongly linked to health outcomes, specific relations may exist for TV viewing and other adverse health behaviors, such as poor diet.

A major strength of our study is that we asked older persons to report the time spent on ten different sedentary activities. The largest amount of time was spent watching TV (3.2 h/d, 32% of total self-reported sedentary time), followed by reading (1.6 h/d) and hobby (1.1 h/d). Few other studies have incorporated sedentary activities other than TV viewing [8-10]. A study among younger adults, aged 20–65 y, found that sedentary activities such as TV viewing, car driving and sitting/talking were performed most [9]. Results for the subgroup of persons aged ≥60 years in the study by Salmon et al. [8] showed that TV viewing (1.7 h/d), reading (0.9 h/d) and socializing (0.7 h/d) were the most common sedentary activities. Information on the specific sedentary activities performed by older persons may support the development of interventions to reduce sedentary time.

In our study, we searched for the best set performing of sedentary activities, with the highest correlation coefficient with total objective sedentary time, which could be used to optimally rank people’s total sedentary time. The best performing set of sedentary activities proved to include six different activities. Excluding four of the ten activities we investigated reduced the total self-reported sedentary time from 10.4 to 8.1 h/d, which was statistically significantly different from the total objective sedentary time of 10.2 h/d (p < 0.01). Although the agreement in absolute sedentary time was thus reduced by excluding the four activities, the correlation with total objective sedentary time increased to 0.46. Thus, if obtaining accelerometry data is not feasible, a questionnaire including these six sedentary activities might enable persons to be ranked as having relatively low and relatively high level of sedentary time, which is often important in epidemiological research. The correlation we found is stronger than previously published associations between self-reported time spent on sedentary activities and accelerometry in adolescents (r = 0.14 [24]) and adults (r = 0.33) [25], but weaker than the associations found in studies among younger adults using a 15-minute interval diary (r = 0.56-0.87) [26] or a two-day time use survey with 5-minute intervals (r = 0.74) [27] to assess self-reported sedentary time. Two recent studies among older adults that compared self-reported sedentary items by questionnaire with accelerometry data found weaker correlations (r = 0.12-0.30) [10,28] than presented in our study. Future studies should confirm whether our set of six sedentary activities is the most suitable set to rank sedentary behavior in older persons. Further studies are also required to identify the optimal subjective methods to estimate sedentary time in young and older persons.

Total sedentary time tended to be different between weekdays and weekend days, as the older persons in our sample spent more time on sedentary behavior during weekend days than on weekdays. This was consistently observed in both the self-reported and objective data. The objective time spent on sedentary activities relative to total accelerometer wear time was statistically significantly higher during the weekend. This suggests that questionnaires should include questions on sedentary time for both weekdays and weekend days to obtain a good estimate of overall sedentary time for an average day. The results of our sensitivity analysis showed that using self-reported information from weekdays only reduced the correlation coefficient from 0.46 to 0.42. Using information from weekend days only caused a much greater reduction of the coefficient (from 0.46 to 0.36). If necessary, questionnaire length could be reduced by referring to weekdays only.

A strength of our study is the use of accelerometry to objectively assess total sedentary time, instead of activity diaries [29,30] or cardio-respiratory fitness [31]. Other strengths are the inclusion of ten different sedentary activities which are often performed by older persons and the inclusion of information from both weekdays and weekend days. Our study sample was comparable to the overall LASA sample with regard to some basic characteristics, with only a significant difference in education level. A potential limitation is that no cross-validation sample was available to confirm whether the set of six sedentary activities is indeed the best to predict total objective sedentary time in older persons, so future studies will have to address this issue. A further limitation is that participants were instructed to report only one sedentary activity if they performed two at the same time. We do not have information on the number of participants who reported multiple simultaneous activities and how this may have impacted on the estimated time spent on each sedentary activity. Further, the accelerometer used in our study detects movement and not posture; the latter function would increase the accuracy to assess sedentary time objectively. Finally, there was a potential bias in the form of a Hawthorne effect, as sedentary behavior levels may have been influenced by wearing the accelerometer.

## Conclusions

In conclusion, older persons in our sample spent 10.2 hours per day on sedentary activities. Although TV watching was the major sedentary activity performed, the time spent watching TV should not be used as an indicator of total sedentary time in older persons. A questionnaire including six different sedentary activities optimally and reliably ranked older persons with regard to sedentary time, and was moderately associated with accelerometry-derived sedentary time. This questionnaire may complement physical activity questionnaires to provide an overall assessment of daily activity across the full range of intensities.

## Competing interests

The authors declare that they have no competing interests.

## Author’s contributions

MV conceptualized the study idea and wrote the article. AK analyzed the data and contributed to drafts of the article. Both authors read and approved the final manuscript.

## Pre-publication history

The pre-publication history for this paper can be accessed here:

http://www.biomedcentral.com/1471-2318/13/80/prepub

## Supplementary Material

Additional file 1 LASA Sedentary Behavior Questionnaire. Click here for file
