# Supplementary material for: Development of a questionnaire to assess sedentary time in older persons – a comparative study using accelerometry
Source: BMC Geriatr. 2013 Jul 30;13:80. doi: 10.1186/1471-2318-13-80 (PMC3733654; doi:10.1186/1471-2318-13-80)
Supplement: Additional file 1 — LASA Sedentary Behavior Questionnaire. [file 1471-2318-13-80-S1.docx]

**Longitudinal Aging Study Amsterdam** (LASA)

Author: Marjolein Visser

**LASA Sedentary Behavior Questionnaire**

The LASA Sedentary Behavior Questionnaire is a self-administered questionnaire to asses sedentary behavior of older persons. The questionnaire consists of 10 items. The average time per 24 hours spend on each individual sedentary activity will be recorded in hours and/or minutes. This will be done for an average weekday as well as for an average weekend day.

| **On average, during a weekday (Monday - Friday), how many hours / minutes in a day (24 hours) do you ……** | | |
| --- | --- | --- |
| 1* | take a nap on a chair or couch?...................................................... | ___ h ____ min |
| 2* | read while being seated or lying down? | ___ h ____ min |
| 3* | listen to music while being seated or lying down? | ___ h ____ min |
| 4* | watch television, video or DVD? | ___ h ____ min |
| 5* | perform a hobby while being seated, such as knitting, doing jigsaw puzzles or playing a music instrument? | ___ h ____ min |
| 6* | talk (in person or on the phone) with friends, family or acquaintances while being seated?................................................. | ___ h ____ min |
| 7 | sit at the computer for work or leisure………………………… | ___ h ____ min |
| 8 | perform sitting activities such as administrative tasks, writing a letter or having a meeting……………………………………………. | ___ h ____ min |
| 9 | sit in car, bus or train…………………………………………… | ___ h ____ min |
| 10 | visit church or (movie) theater……………………………………. | ___ h ____ min |

Instruction study participant:

*The next questions refer to the time you spend sitting or lying down during a full day (24 hours). Could you first respond to the questions for an average weekday (Monday-Friday) and then for an average weekend day (Saturday and Sunday)? If you do not perform an activity, please write down ‘0’ (zero).*

*PLEASE NOTE: if you perform two activities at the same time, for example listening to music while knitting, please report only one of the two activities. You can choose yourself for which activity you report this time.*

| **On average, during the weekend (Saturday and Sunday), how many hours / minutes in a day (24 hours) do you ……** | | |
| --- | --- | --- |
| 1* | take a nap on a chair or couch?...................................................... | ___ h ____ min |
| 2* | read while being seated or lying down? | ___ h ____ min |
| 3* | listen to music while being seated or lying down? | ___ h ____ min |
| 4* | watch television, video or DVD? | ___ h ____ min |
| 5* | perform a hobby while being seated, such as knitting, doing jigsaw puzzles or playing a music instrument? | ___ h ____ min |
| 6* | talk (in person or on the phone) with friends, family or acquaintances while being seated?................................................. | ___ h ____ min |
| 7 | sit at the computer for work or leisure………………………… | ___ h ____ min |
| 8 | perform sitting activities such as administrative tasks, writing a letter or having a meeting……………………………………………. | ___ h ____ min |
| 9 | sit in car, bus or train…………………………………………… | ___ h ____ min |
| 10 | visit church or (movie) theater……………………………………. | ___ h ____ min |
